# Genotyping‐free parentage assignment using RAD‐seq reads

**DOI:** 10.1002/ece3.6503

**Published:** 2020-06-30

**Authors:** Shi‐Yi Chen, Cao Li, Zhihao Luo, Xiaowei Li, Jia Gan, Xianbo Jia, Song‐Jia Lai, Wei Wang

**Affiliations:** ^1^ Farm Animal Genetic Resources Exploration and Innovation Key Laboratory of Sichuan Province Sichuan Agricultural University Chengdu China; ^2^ Longri Breeding Farm of Sichuan Province Hongyuan China; ^3^ Sichuan Animal Science Academy Chengdu China

**Keywords:** genome heterozygosity, likelihood ratio, Mexican gray wolf, yak

## Abstract

Parentage assignment is defined as the identification of the true parents of one focal offspring among a list of candidates and has been commonly used in zoological, ecological, and agricultural studies. Although likelihood‐based parentage assignment is the preferred method in most cases, it requires genotyping a predefined set of DNA markers and providing their population allele frequencies. In the present study, we proposed an alternative method of parentage assignment that does not depend on genotype data and prior information of allele frequencies. Our method employs the restriction site‐associated DNA sequencing (RAD‐seq) reads for clustering into the overlapped RAD loci among the compared individuals, following which the likelihood ratio of parentage assignment could be directly calculated using two parameters—the genome heterozygosity and error rate of sequencing reads. This method was validated on one simulated and two real data sets with the accurate assignment of true parents to focal offspring. However, our method could not provide a statistical confidence to conclude that the first ranked candidate is a true parent.

## Graphical abstract

A new likelihood‐based method for parentage assignment has been proposed using RAD‐seq reads in this study. Due to not depending on the genotype data and prior information of population allele frequencies, it is expected that this method can be applied more widely.

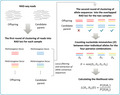

## 1 INTRODUCTION

Parentage analysis aims to identify individual pedigree relationships using codominant molecular markers. It has been commonly involved in zoological, ecological, and agricultural studies (Huang, Mi, Dunn, Wang, & Li, 2018). A common practice of parentage analysis is achieved through one‐by‐one exclusion of nonparentage individuals and/or probability‐based assignment of the parentage individuals (Jones, Small, Paczolt, & Ratterman, 2010). Among a set of candidate parents for one focal offspring, each of them could be excluded by observing one or more loci with Mendelian inconsistencies. The latter approach could be further classified into categorical and fractional allocations, which rely on the statistical estimations of individual likelihoods or Bayesian posterior probabilities under parentage and nonparentage hypotheses. Although the molecular markers used have been successively updated from allozymes, microsatellites, to single nucleotide polymorphisms (SNPs) during the past three decades, the theoretical basis of parentage analysis has not deviated from obeying Mendel's law (Flanagan & Jones, 2019).

Microsatellites are the first‐generation DNA markers that have been practically widely used for parentage analysis due to the high polymorphism information content, abundant distribution, and convenience in genotyping (Selkoe & Toonen, 2006; Webster & Reichart, 2005). Because it has become economically feasible to obtain tens of thousands of genome‐wide SNPs using oligonucleotide arrays and high‐throughput sequencing (HTS) approaches, SNP markers are expected and also have already been proven to be a reliable alternative to microsatellites (Andrews et al., 2018; Hayes, 2011; Heaton et al., 2014; Strucken et al., 2014). Both strengths and weaknesses had been systematically compared between microsatellites and SNPs in the context of parentage analysis (Fernández et al., 2013; Tokarska et al., 2009). It has been generally agreed that 100–500 SNPs are sufficient to ensure successful parentage analysis in most situations (Flanagan & Jones, 2019). Recently, an R package (Huisman, 2017) and bioinformatic pipeline (Andrews et al., 2018) have been successfully developed for specifically addressing the SNP‐based parentage analysis.

Although HTS approaches provide a promising strategy for parentage analysis, almost all of existing methods follow the same theoretical logic to microsatellite‐based methods because SNPs must be called and genotyped prior to statistical inferences (Andrews et al., 2018; Thrasher, Butcher, Campagna, Webster, & Lovette, 2018). Also, the prior information of SNP allele frequencies in the studied populations is required for calculating likelihoods. In practice, it would be a time‐consuming process to genotype SNPs especially when a large number of individuals are involved, and the rigorous requirement for providing prior allele frequencies may limit the applicability of these previous methods especially in less‐studied populations. To address the two drawbacks, an alternative idea is to directly employ the entire DNA fragments of short HTS reads as molecular markers and therefore estimate the likelihoods by an allele frequency‐free method. Waples, Albrechtsen, and Moltke (2019) proposed an inference method of close familial relationships without requiring allele frequency information for the genotyped SNPs. The restriction site‐associated DNA sequencing (RAD‐seq) is a widely used HTS approach for discovering genome‐wide SNPs that could be efficiently used for parentage analysis (Miller, Dunham, Amores, Cresko, & Johnson, 2006). Recently, new methods of parentage analysis have been specifically developed mainly focusing on RAD‐seq data (Dodds et al., 2019; Whalen, Gorjanc, & Hickey, 2019). Another potential advantage of RAD‐seq is to generate a large number of short DNA fragments at high coverage that overlap well among all the sequenced individuals. Therefore, it is anticipated that these DNA fragments can be directly compared for parentage analysis without requiring the SNP genotyping in advance.

In the present study, we provided an alternative method of likelihood‐based parentage assignment that directly compares RAD‐seq reads and no longer depends on genotype data and prior information of population allele frequencies. This method was successfully validated on one simulated data set of cattle (*Bos taurus*) and two real data sets of Mexican gray wolf (*Canis lupus baileyi*) and yak (*B. grunniens*).

## 2 MATERIALS AND METHODS

### 2.1 Ethics statement

Yak blood samples involved in the present study were collected by veterinarians for annual health inspection, which means that no ethical approval was required.

### 2.2 Algorithmic overview

**FIGURE 1:**
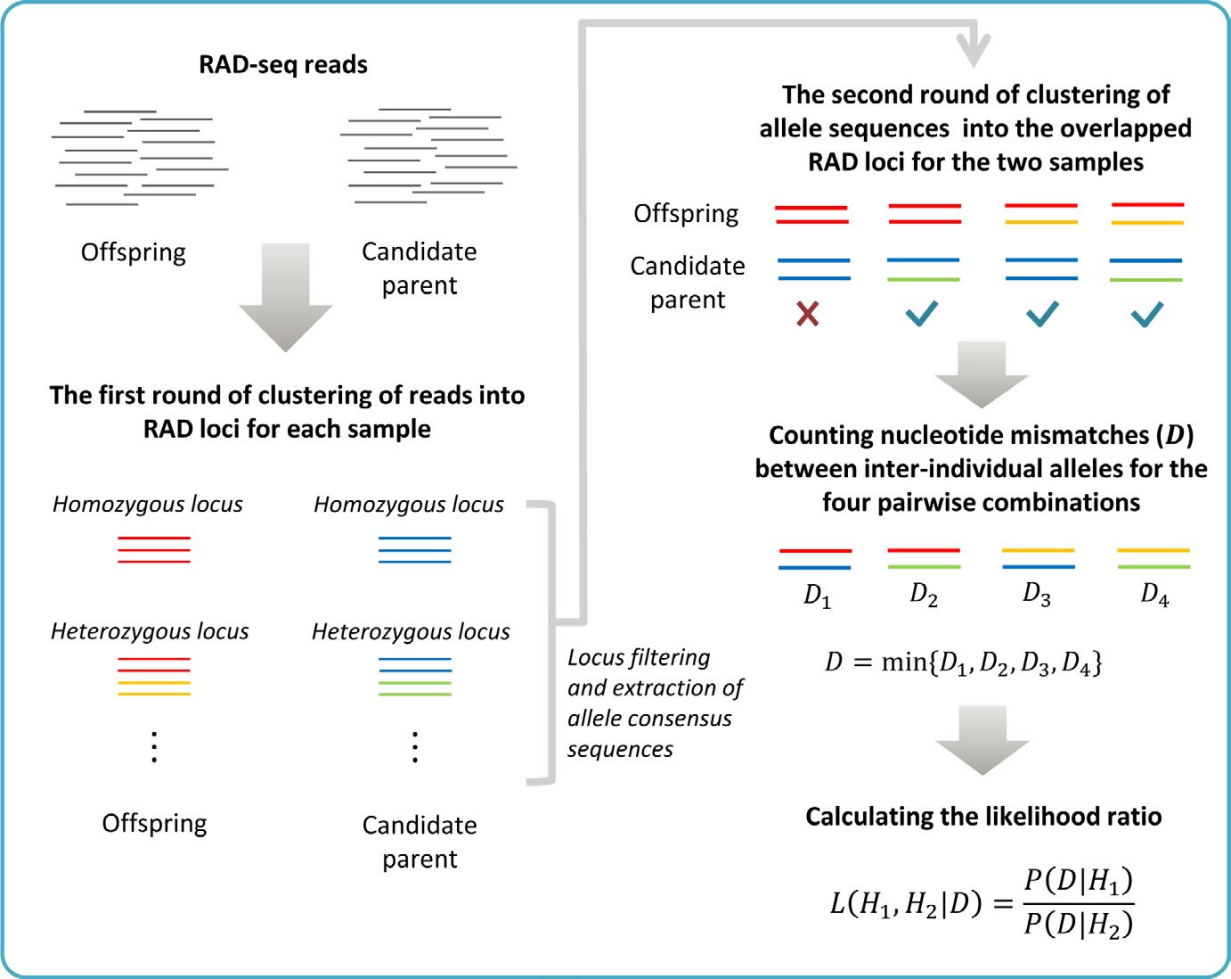
Schematic illustration of algorithm. Gray short lines represent the initial RAD‐seq reads after quality filtering. Reads from one RAD locus are stacked together and colored for different alleles. Note: The different colors of inter‐individual alleles only indicate their origins; that is, their DNA sequences could be 100% identical with 0 nucleotide mismatch

The basic logic of our algorithm is a two‐round de novo clustering of RAD‐seq reads and is schematically illustrated in Figure 1. The first round is conducted independently on each sample for clustering the RAD‐seq reads into all possible RAD loci that would be homozygous or heterozygous. After filtering low‐quality loci, allele consensus sequences are extracted for the two compared individuals (i.e., the focal offspring and a candidate parent) and subjected to the second round of clustering with the aim of determining their overlapped RAD loci. Therefore, this procedure of two‐round clustering will produce the comparable RAD loci for a pair of focal offspring–candidate parent. The minimum number of nucleotide mismatches (D) could be obtained among the four combinations of inter‐individual alleles at every RAD locus, which is finally used for calculating likelihood ratio of parentage assignment.

### 2.3 Sequence clustering and locus filtering

We cluster sequences using the ustacks module in Stacks toolset (Paris, Stevens, & Catchen, 2017; Rochette, Rivera‐Colón, & Catchen, 2019), which has two parameters of the minimum read depth in supporting an effective allele (‐m) and the maximum number of nucleotide differences between alleles (‐M) that could be tuned for adaptively processing the two‐round clustering of both RAD‐seq reads and allele consensus sequences. For the first‐round clustering of RAD‐seq reads, the parameters of ‐m and ‐M could be set for controlling the confidence of a constructed RAD locus and sequencing errors of RAD‐seq reads, respectively. Meanwhile, the RAD loci that have more than two alleles are first filtered out before extracting allele consensus sequences.

During the second round of clustering, the parameter of ‐m must be adaptively set to 1 because only one consensus sequence was retained for each allele. As the sequencing errors had been eliminated after the first round of clustering, the parameter of –M could be conservatively set to a relatively large value, such as 4 or higher, for clustering the inter‐individual alleles. After obtaining the overlapped RAD loci, the loci that are simultaneously homozygous in both the focal offspring and a compared candidate parent are discarded to ensure that all the finally used RAD loci are actually derived from autosomes. To guarantee the direct comparability for multiple candidate parents, only the intersected RAD loci among one focal offspring and all its candidate parents will be used for calculating the likelihood ratios.

### 2.4 Calculation of likelihood ratios

In the absence of sequencing error and germ‐line mutation, an offspring and its true parent must share at least one allele with identical nucleotide sequence at each overlapped RAD locus; however, a pair of identical alleles could be also derived from any two unrelated individuals due to short sequence fragment and low genome heterozygosity. Because it is unknown which allele has been parentally transmitted at each RAD locus, we select the most likely inter‐individual allele pair among the four possible combinations by the minimum number of nucleotide mismatches (Figure 1). For this candidate allele pair with an observed number of nucleotide mismatches (D), we calculate the likelihood ratio under parentage to nonparentage relationships, which are modeled by both the sequencing error rate and genome heterozygosity.

Let r, h, and L represent the sequencing error rate, genome heterozygosity, and sequence length of RAD‐seq reads, respectively. The distribution of D is determined by the three parameters of r, h, and L and could be described as a Poisson random variable because of the very low values of both r and h. Under the hypothesis $H_{1}$ that the offspring is compared with its true parent, the probability of observing D is only determined by r and L and can be expressed:

$$P\left(D|H_{1}\right)=\frac{{\left(2Lr\right)}^{D}}{D!}e^{-2Lr}$$

In this formula, the sequence length should be doubled (2L) because a sequencing error could be observed on any of the two alleles. Under the hypothesis $H_{2}$ that the offspring is compared with an unrelated random individual, the probability of observing D will be additionally determined by h and can be expressed:

$$P\left(D|H_{2}\right)=\frac{{\left[2L\left(r+h\right)\right]}^{D}}{D!}e^{-2L(r+h)}$$

Here, the likelihood ratio under $H_{1}$ and $H_{2}$ hypotheses is given as:

$$L\left(H_{1},H_{2}|D\right)=\frac{P(D|H_{1})}{P(D|H_{2})}$$

Finally, the likelihood ratios of all RAD loci are multiplied to obtain the combined likelihood ratio, which is further transformed into the LOD score by natural logarithm (Marshall, Slate, Kruuk, & Pemberton, 1998).

### 2.5 Simulated and real data sets

We first simulated a population data of domestic cattle for validating our method. The QMSim tool (Sargolzaei & Schenkel, 2009) was used to produce the genomic data by mimicking a real population. A historical population of 100 generations was constructed with a decreasing effective population size from 5,000 in the first generation to 500 in the 100th generation; within each generation, equal numbers of male and females were randomly mated. Beginning from the last generation of the historical population, 20 males and 50 females were randomly selected for producing a current population with a total of 10 generations. The mating procedure for the current population was designed to minimize inbreeding, and the litter size was set to 2. Based on the recent assembly of cattle reference genome (ARS‐UCD1.2), all 29 real autosomes were employed for producing the genome‐wide SNPs. All SNPs were randomly distributed through all chromosomes with a mean density of one SNP per 1 kb region. Based on the created population and genome‐wide SNPs, RAD‐seq reads were then simulated for all 100 animals of both 9th and 10th generations in the current population using RADinitio tool (Rivera‐Colón, Rochette, & Catchen, 2019). During this process, the genome DNA was digested with a single enzyme of *SbfI* and subjected to sequencing at 20X coverage.

In addition to the simulated data set above, our method was further validated on two real data sets. This first was a published RAD‐seq data set of Mexican gray wolf, which was used for testing bioinformatic pipeline of SNP‐based parentage assignment (Andrews et al., 2018). From their initial samples, we randomly selected 28 individuals consisting of 12 offspring (pups), six true parents, and 10 unrelated candidate parents (Table 1). The second was a newly sequenced data set of 14 domestic yaks, which included four parent–offspring pairs and six unrelated candidate parents. These blood samples of yaks were collected in Hongyuan County of Sichuan province and subjected to RAD‐seq (Baird et al., 2008). Briefly, ~1 μg genomic DNA per sample was used to construct sequencing libraries according to the recommended pipeline, which mainly consists of double digestion by *RsaI* and *HaeIII* (New England Biolabs), ligation of adapter sequences, and sample pooling. Subsequently, DNA fragments with the 450–480 bp in length were selected and sequenced on Illumina HiSeq™ 2000 platform to generate 100 bp paired‐end reads (Biomarker Technologies Corporation).

**TABLE 1:** Overlapped RAD loci of each offspring with all candidate parents for the two real data sets

Data sets	Offspring		Compared with all candidate parents (*N*)		
	IDs	RAD loci (*N*)	Minimum	Maximum	Intersected
Mexican gray wolf	W1349	195,435	31,919	44,216	16,071
	W1350	177,804	25,605	37,521	12,054
	W1352	183,240	26,444	38,419	12,071
	W1354	175,553	24,484	36,086	11,082
	W1383	185,087	38,937	49,437	23,229
	W1385	184,255	27,743	39,077	13,265
	W1390	198,726	56,525	68,760	36,334
	W1392	190,580	28,099	40,975	13,255
	W1398	195,602	35,354	47,451	18,418
	W1439	185,247	28,896	39,641	14,365
	W1487	181,027	27,403	39,460	13,540
	W1552	190,702	35,408	47,568	19,200
Yak	Y203	230,393	9,450	36,762	2,602
	Y204	275,720	8,939	37,117	2,177
	Y205	378,758	11,162	43,901	2,482
	Y207	338,499	11,688	39,375	2,687

### 2.6 Data analyses

All raw RAD‐seq reads, including the simulated and actually sequenced, were first subjected to quality control (QC) using the fastp tool (Chen, Zhou, Chen, & Gu, 2018), in which the low‐quality reads were filtered out by any one of the three criteria: (a) reads containing adapter sequences or ambiguous bases, (b) reads containing low‐quality bases (*Q*_phred_ value <15) more than 40% of the total length, and (c) reads with <40% complexity defined as the percentage of base that is different from its next base. After potential PCR duplicates were removed using Stacks toolset (Rochette et al., 2019), all analysis steps were conducted according to our method described above, and the ustacks parameters of ‐m 3 and ‐M 2 were set for the first round of clustering, and ‐m 1 and ‐M 4 for the second round of clustering. When obtaining the variable of D for the candidate parent–offspring pairs, the likelihood ratios and LOD scores were calculated using the parameters of r = 0.0024 and h = 0.001 for all three data sets (Eck et al., 2009; Pfeiffer et al., 2018; Qiu et al., 2012). Besides that the sequence clustering was performed using Stacks toolset (Rochette et al., 2019), all processing steps were conducted by our custom Python scripts with source codes freely available at https://github.com/chengroup/PPA.

## 3 RESULTS

### 3.1 Validation in simulated data

The simulated population genomic data of cattle included 2,484,887 SNPs and consisted of 100 parents in the 9th generation and 100 offspring in the 10th generation. A total of 501 million high‐quality RAD‐seq reads were simulated and subjected to sequence clustering with the forward reads. We successfully constructed on average 123,632 RAD loci per sample (ranging from 123,563 to 123,697), from which the ambiguous and homozygous RAD loci were further removed. After each offspring was compared with its all candidate parents for producing their intersected RAD loci, we calculated the likelihood ratios and LOD scores for every candidate offspring–parent pair. A total of 50 offspring were randomly selected for parentage assignment, and each of them had 30 candidate parents including the two true parents and 28 randomly selected nonparentage individuals.

**FIGURE 2:**
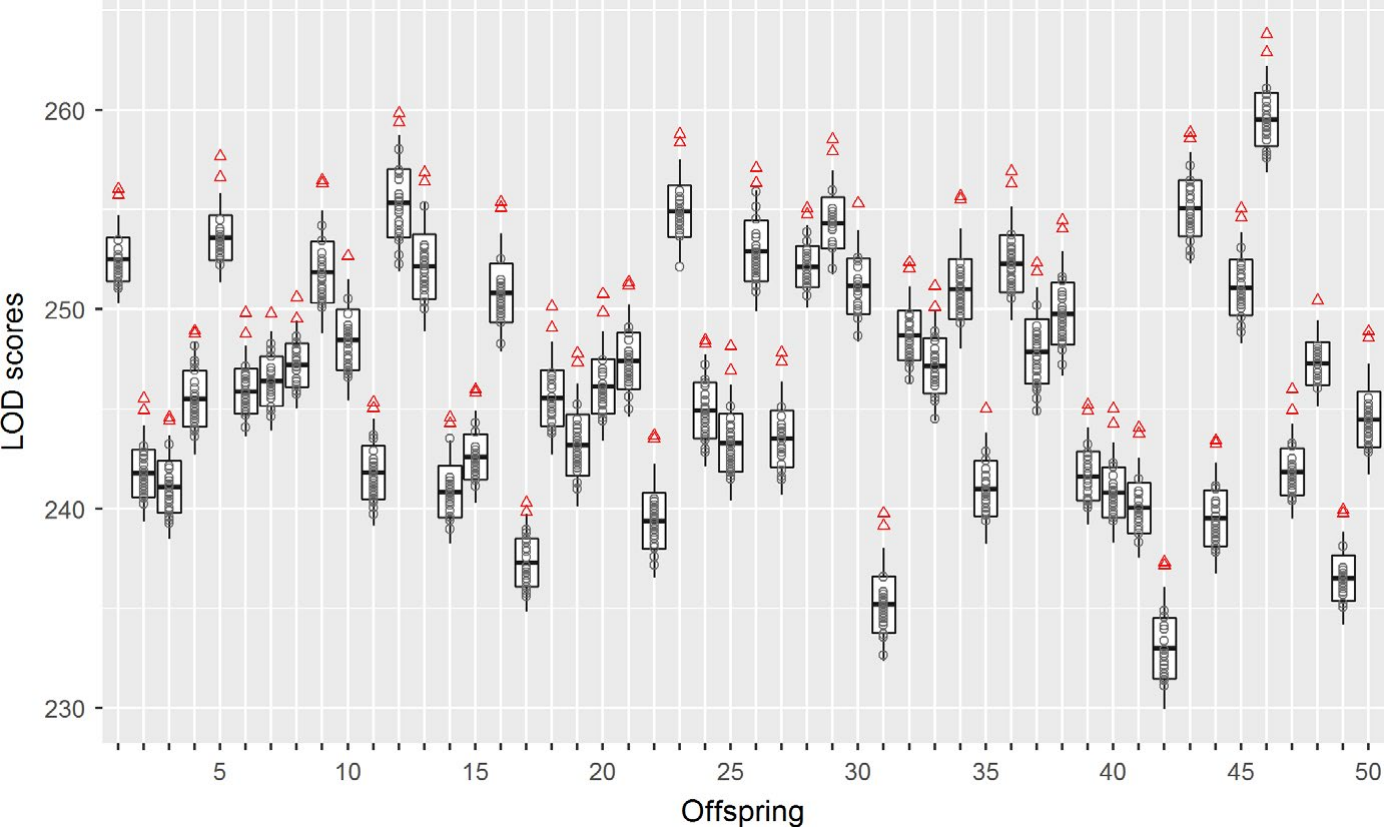
Distribution and comparison of LOD scores in the simulated data set. For each offspring (a total of 50 offspring on the *X*‐axis), the distribution of LOD scores is shown as box‐and‐whisker plots, which indicate the mean (line in the box), mean ± SD (box extension), and mean ± 2 × SD (whiskers), respectively. Subsequently, all LOD scores are individually plotted by the gray circle (nonparentage individuals) and red triangle point‐up (true parents). SD: standard deviation

Because up to 30 LOD scores were obtained for each offspring in this simulated data set, we first investigated their overall distribution pattern (Figure 2). These LOD scores were ranged from 231.1 to 263.8 with the obvious variation among offspring. Within each offspring, however, the LOD scores of all candidate parents were much more consistent with each other. By selecting the highest two LOD scores, the true parents were correctly identified for all 50 offspring. For every offspring, both true parents had LOD scores out of two standard deviations (SDs) interval and could be completely distinguished from the 28 nonparentage individuals. On the other hand, all LOD scores of nonparentage individuals fell within the two SDs only with one exception (14# offspring), in which one candidate had a slightly higher LOD score than this threshold value.

### 3.2 Parentage assignment of Mexican gray wolf

Because the data set of Mexican gray wolf was produced by single restriction enzyme of *SbfI*, only the forward reads were used for clustering of RAD loci and calculation of likelihood ratios. An average of 7.15 million raw (ranging from 2.75 to 28.29) and 5.93 million clean (ranging from 2.47 to 19.86) reads were initially obtained for the 28 samples, respectively (Table S1). Subsequently, the high‐quality reads of every sample were independently clustered into from 168,625 to 213,441 RAD loci. About 1.56% of them had more than two alleles and therefore were excluded from the following analyses.

**FIGURE 3:**
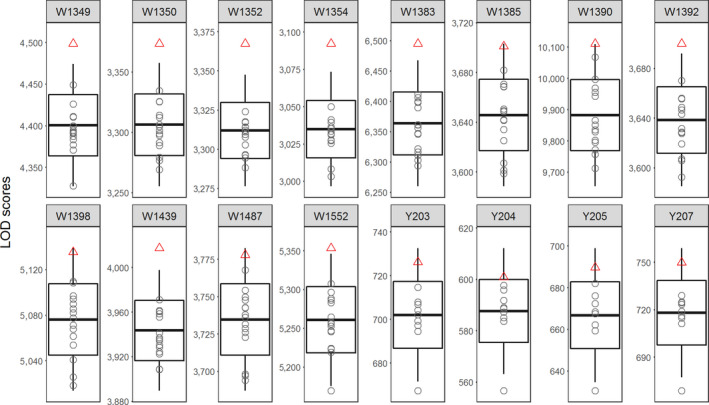
Parentage assignment of Mexican gray wolf and yak. This multi‐panel plot successively consists of 12 offspring of Mexican gray wolf (prefixed by letter W) and four offspring of yak (prefixed by letter Y). The plotting methods are same to Figure 2

For each offspring–parent comparison, the minimum and maximum numbers of the overlapped RAD loci ranged from 24,484 to 56,525 and from 36,086 to 68,760, respectively (Table 1). Among all pups, we obtained from 11,082 to 36,334 comparable RAD loci that had been intersected among one focal offspring and all its candidate parents, that is, the numbers of intersected RAD loci in Table 1. All these comparable RAD loci were used for calculating the likelihood ratio and LOD score for each candidate offspring–parent pair (Table S2). The LOD scores ranged considerably, from 3,003 to 10,111 among the 12 pups (Figure 3), and depended on the numbers of RAD loci used for each offspring. We found that the true parents of all focal pups were correctly ranked in the first position according to their LOD scores. Furthermore, nine and three true parents had the LOD scores out of and close to two SDs, respectively.

**FIGURE 4:**
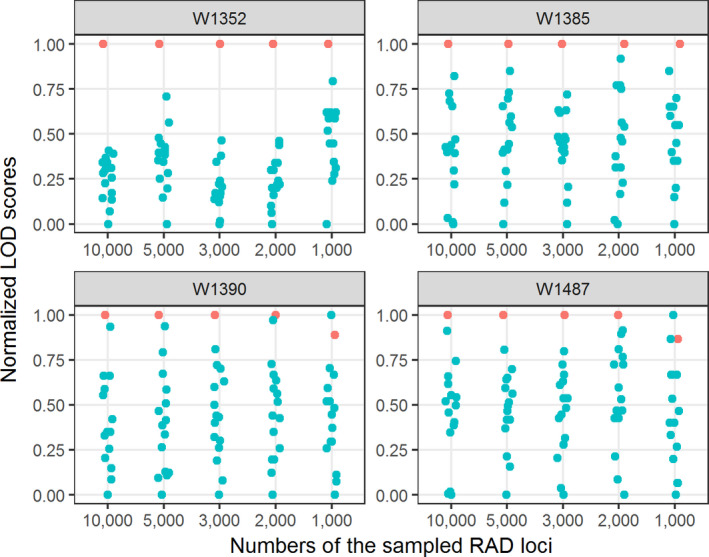
Parentage assignment by the subsampled RAD loci for four offspring of Mexican gray wolf. Each point represents a candidate parent. The true parents and nonparentage individuals are colored in red and blue, respectively

Due to the fact that more than ten thousand comparable RAD loci were obtained for all offspring in this real data set, we preliminarily investigated the accuracy in parentage assignments using the randomly reduced numbers of RAD loci from 10,000 to 1,000 loci (Figure 4). By analyzing four pups that had the highest and lowest differences in LOD scores (Δ) between true parent and the next nonparentage individual, we found that the true parents could be correctly assigned when more than 2,000 intersected RAD loci were used. Two pups of W1390 and W1487 were falsely assigned by nonparentage individuals when the RAD loci were reduced to 1,000. On the whole, Δ values varied obviously along with the used numbers of RAD loci.

### 3.3 Parentage assignment of yak

We produced 109.18 million raw reads for the 14 sequenced yaks, from which an average of 4.71 million clean reads per sample were finally obtained (Table S1). The intra‐individual clustering of reads initially resulted in from 231,693 to 420,491 RAD loci among all samples. After filtering out the ambiguous RAD loci (~0.45%), there were from 230,393 to 378,758 RAD loci for the four calves that were then compared with the 10 candidate parents (Table 1). Among all the offspring–parent comparisons, the minimum and maximum numbers of overlapped RAD loci were 8,939 and 43,901, respectively. Finally, more than 2,000 intersected RAD loci (ranging from 2,177 to 2,687) were produced and used for calculating likelihood ratios and LOD scores for each candidate offspring–parent pair (Table S3). The LOD scores ranged from 557 to 745, by which the true parents were correctly assigned to all calves (Figure 3). However, LOD scores of all true parents fell outside one *SD* but did not exceed the two SDs.

## 4 DISCUSSION

As one of the most successful applications of DNA‐based techniques during the past decades, parentage analysis has been extensively used for addressing theoretical and practical questions in zoological, ecological, and agricultural studies (Hayes, 2011; Heaton et al., 2014; Strucken et al., 2017). For example in farm animals, accurate identification of true parents is vital for the genetic evaluation and individual selection in multi‐sire pasture‐based systems (Wang et al., 2012). Also, many efforts have been devoted to improve the cross‐population comparability of likelihood‐based parentage analysis, such as the International Society for Animal Genetics having proposed reference panels of parentage analysis for both microsatellites and SNPs (ISAG, www.isag.us). However, two drawbacks remain to be addressed with respect to the likelihood calculation of existing methods. First, the prior information of allele frequencies of DNA markers, which are required for calculating the likelihood ratio, is always unavailable, and not even approximately for less‐studied or genetically distant populations. Furthermore, population allele frequencies cannot be accurately calculated de novo when only a relatively small sample size is involved. Recently, an allele frequency‐free method was proposed to infer pairwise relatedness among these close familial members based on the allele identity‐by‐state status (Waples et al., 2019). The second issue is that the efficient genotyping for a predefined set of DNA markers would be a time‐consuming task. Accordingly, we proposed and validated an alternative method of parentage assignment in the present study, which does not depend on having explicit genotype data or prior information of population allele frequencies.

In addition to application for discovering the genome‐wide SNPs, the RAD‐seq approach has another advantage of producing a large number of inter‐individual overlapped DNA fragments in high coverage (Miller et al., 2006). Because these short DNA fragments on the sequenced RAD loci could be directly compared among individuals, we theoretically expected that they contain enough genetic information in context of parentage analysis. Recently, two new methods have been developed for parentage analysis using RAD‐seq and similar data, which primarily addressed the issues of low accuracy of SNP genotyping and high missing rate of genotypes that would result from low and/or highly variable sequencing coverage among individuals (Dodds et al., 2019; Whalen et al., 2019). Instead of subjecting RAD‐seq reads to SNP genotyping in advance, inter‐individual allelic nucleotide mismatch at a RAD locus could be directly observed and used for calculating the likelihood ratio of parentage analysis. More importantly, we could easily model this variable by two relatively constant parameters—the error rate of sequencing reads and genome heterozygosity. The average error rate of 0.24 ± 0.06% per base was systematically estimated for HTS approaches (Pfeiffer et al., 2018). The estimated genome heterozygosity slightly varied around 0.1% (i.e., about one SNP per 1,000 bp) for main farm animals, such as cattle (Eck et al., 2009), sheep (Jiang et al., 2014), and yak (Qiu et al., 2012).

Although numerous tools have been developed for DNA marker‐based parentage analysis, such as the widely used CERVUS (Kalinowski, Taper, & Marshall, 2007) and COLONY (Jones & Wang, 2010), there would be no significant difference on detection accuracy due to their similar theoretical basis. Also, we proposed the new method of parentage assignment in the present study mainly because it employed an alternative inference algorithm in comparison with existing methods. In other words, we do not think that our method is superior to others in terms of assignment accuracy but can be applied more widely. In addition to RAD‐seq data, our method could be applied to other HTS genomic data if there are enough DNA fragments that could be overlapped and compared between a focal offspring and its candidate parents. Despite our conservative estimate that at least 2,000 RAD loci (with a nonmissing genotype for the offspring and all its candidate parents) are required for obtaining reliable assignment of true parents, such numbers of RAD loci could be easily obtained by RAD‐seq and other similar approaches due to the use of restriction enzymes. Additionally, a relatively high sequencing coverage would be helpful to avoid the occurrence of null alleles (Dakin & Avise, 2004).

Although one‐by‐one exclusion of nonparentage individuals is feasible and also useful in some cases (Hayes, 2011), the likelihood‐based approaches have been more widely used for parentage analysis due to their statistical foundations and easy incorporation of genotyping error rates (Jones et al., 2010). More importantly, the statistical confidence could be derived with respect to the most likely true parent that holds the highest likelihood ratio or LOD score (Marshall et al., 1998). Despite accurately assigning the true parents to every focal offspring according to the LOD scores in the present study, our method remains hard to make a statistical conclusion about whether the first ranked candidate is a true parent. We empirically found that the true parents would have the significantly deviated LOD scores by two or one SDs. When it is uncertain whether the true parent has been included into the candidate set, additional information would be required to make the conclusion of parentage assignment.

## CONFLICT OF INTEREST

None declared.

## AUTHOR CONTRIBUTIONS

**Shi‐Yi Chen:** Conceptualization (equal); formal analysis (equal); writing – original draft (equal); writing – review & editing (equal). **Cao Li:** Conceptualization (equal); formal analysis (equal); writing – review & editing (equal). **Zhihao Luo:** Resources (equal). **Xiaowei Li:** Resources (equal). **Jia Gan:** Resources (equal). **Xianbo Jia:** Resources (equal). **Song‐Jia Lai:** Resources (equal). **Wei Wang:** Formal analysis (equal); resources (equal); writing – review & editing (equal).

## Supporting information

Table S1‐S3

## Data Availability

The RAD‐seq data of yaks have been deposited to NCBI Sequence Read Archive (SRA) under accession of SRP234112.
